# Real World Evidence of Wearable Smartbelt for Mitigation of Fall Impact in Older Adult Care

**DOI:** 10.1109/JTEHM.2023.3256893

**Published:** 2023-03-15

**Authors:** Rebecca J. Tarbert, Wamis Singhatat

**Affiliations:** Active Protective Technologies, Inc Fort Washington PA 19034 USA

**Keywords:** Falls, fall injury, hip injury, older adults, wearable technology, intervention

## Abstract

Structured Abstract Falls with major injuries are a devastating occurrence for an older adult with outcomes inclusive of debility, loss of independence and increased mortality. The incidence of falls with major injuries has increased with the growth of the older adult population, and has further risen as a result of reduced physical mobility in recent years due to the Coronavirus pandemic. The standard of care in the effort to reduce major injuries from falling is provided by the CDC through an evidence-based fall risk screening, assessment and intervention initiative (STEADI: Stopping Elderly Accidents and Death Initiative) and is embedded into primary care models throughout residential and institutional settings nationwide. Though the dissemination of this practice has been successfully implemented, recent studies have shown that major injuries from falls have not been reduced. Emerging technology adapted from other industries offers adjunctive intervention in the older adult population at risk of falls and major fall injuries. Technology in the form of a wearable smartbelt that offers automatic airbag deployment to reduce impact forces to the hip region in serious hip-impacting fall scenarios was assessed in a long-term care facility. Device performance was examined in a real-world case series of residents who were identified as being at high-risk of major fall injuries within a long-term care setting. In a timeframe of almost 2 years, 35 residents wore the smartbelt, and 6 falls with airbag deployment occurred with a concomitant reduction in the overall falls with major injury rate.

## I. Introduction

Falls and injuries from falls is a serious challenge for the older adult population and the US healthcare system. According to the CDC, 1 out of 4 older adults will suffer at least one fall per year. In fact, 1 in 5 of those falls will result in a serious injury leading to over 3 million emergency department visits annually. One of the most serious and the costliest injuries from a fall is a hip fracture. Of falls that occur in the older adult US population annually, an average of 300,000 result in a hip fracture [1] The numbers are anticipated to rise with the growing population of older adults worldwide and have further increased as a result of isolation precautions related to the Coronavirus pandemic [2] Reduced mobility offerings associated with infection control measures have resulted in a reduction of engagement in physical activity and a further increase in fall rates [3] The need to address fall injury risk and injury prevention in the older adult population has long been the focus of clinical practice, based upon the American Geriatrics Society and British Geriatrics Society Clinical Practice Guidelines released in 2010 [4] These guidelines are the foundation of the CDC’s STEADI (Stopping Elderly Accidents and Deaths Initiative), which has been incorporated into Medicare Wellness Visit assessments and is the standard of care for fall injury risk across settings. The STEADI includes the screening, assessment and intervention for identifying fall risk and is personalized to the older adult to address the root causes of the risk of fall and fall injury [5] Unfortunately, several recent randomized clinical trials have revealed that interdisciplinary care management utilizing the STEADI with and without enhanced fall prevention efforts did not significantly reduce the rates of fall injuries in the targeted population [6], [7], [8] Falls with major injuries are specifically measured in older adult care settings such as skilled nursing facilities for reporting publicly within the Medicare 5-Star Rating Program. National averages for falls in skilled nursing facilities are recorded at 100–200 annually for a census of 100. In these settings, serious injuries resulting from falls average 10-20% of those falls, with 2-6% of them resulting in a fracture and 7.22 hip fractures per year per facility [9], [10]

**FIGURE 1.**
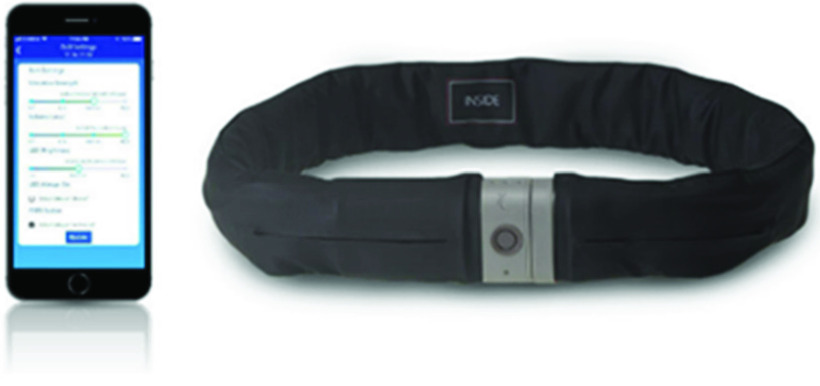
Tango belt and companion mobile app.

Innovative approaches to mitigation of fall impact for the at-risk older adult population are needed to support the standard of care for screening, assessment, and intervention. Emerging wearable technologies include a smartbelt for attenuating ground-impact force due to a serious hip-impacting fall (the Tango Ⓡ Belt, Active Protective Technologies, Inc.). Incorporating core technologies that have been adapted from the smartphone and automotive industries, the smartbelt uses a 9-axis inertial measurement unit and proprietary algorithms to determine if the wearer is experiencing a serious hip-impacting fall in progress and automatically deploys an automotive-grade single-use cold-gas airbag around the hips to attenuate ground-impact forces on the body. The smartbelt also utilizes a companion application that allows for connecting the device to WiFi to view usage metrics including overall compliance and daily wear hours, and also enables communication capabilities. The detection of falls congruently triggers alerting to designated care team members to indicate the need for assistance. The smartbelt is powered by a lithium-ion battery that requires recharging every 3–4 days of continuous use. (See Figure 1.) The smartbelt periodically pushes usage information to a proprietary cloud-based platform, and the companion app pulls key usage metrics, such as daily hours of wear and overall compliance, from the cloud via WiFi to display within the app to facilitate oversight by care staff.

The aim of this descriptive study was to assess the real-world performance of the smartbelt in a case series incorporating the device into the care of older adults at risk of falls and major fall-related injuries in a long-term care setting.

## II. Methods and Procedures

The smartbelt device was to be offered to older adult care settings as a part of fall management programming to establish real-world evidence on feasibility and performance. A long-term care facility with a 250-patient average census agreed to introduce the smartbelt as an adjunct to existing fall injury mitigation measures. Falls management in the center included the use of the STEADI-based MDS 3.0 (Minimum Data Set quarterly resident care assessment tool) for fall risk identification, fall rounds held 3 times/week to offer interdisciplinary care planning for those residents designated to be at risk of falls, and resulting fall risk mitigation strategies. The utilization of Beers criteria for at-risk medication review was applied for all residents in the care setting. Physical and Occupational therapies were prescribed for those demonstrating fall risk with strength, balance and gait impairments. Additionally, pressure sensors to alert for unaided transfer attempts, floor mats and passive hip pads were offered in care plans of those identified as being at risk of falls with major injury. To determine candidates for the smartbelt device, the falls management team was to utilize the STEADI algorithm to identify residents who were at risk for falls by screening for fall history and fear of falling, assessing for the ability to initiate self-transfer/ambulation, and assessing modifiable risk factors, such as gait, strength, balance, blood pressure, and visual acuity. Candidates at risk of falls were to be additionally screened for either an osteoporosis diagnosis or a history of fragility fracture to identify those at risk of major fall-related injury.

**FIGURE 2.**
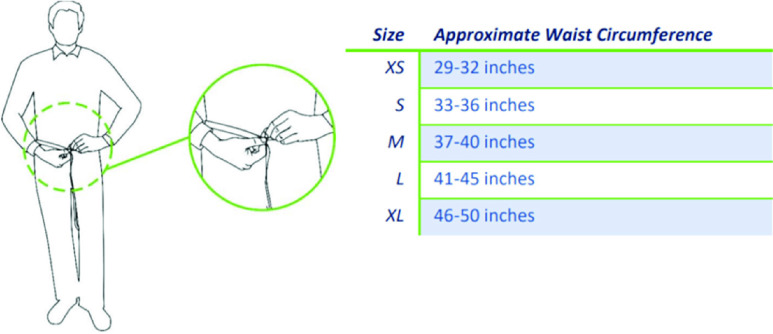
Smartbelt sizing.

Implementation of the smartbelt for each candidate was to be initiated with a physician’s order for the device to be worn up to 24 hours/day or with a specific wear timeframe dependent upon the resident’s care schedule in the facility. Device removal was to be indicated during resident bathing, device charging and as requested by the resident. Upon obtaining consent from the resident or, when applicable, their healthcare POA (Power of Attorney) to use the device, the application of the smartbelt was to be written into the resident’s care plan. Measurement of waist size determined which of the five offered sizes of the smartbelt was to be appropriate. (See Figure 2.) Based on the center’s expected recruitment of 10 residents, an initial inventory of 10 devices was to be connected to the facility’s WiFi upon delivery and assigned to consented residents via room number using the smartbelt companion app.

Performance of the smartbelt was to be assessed by measuring implementation (defined as the percentage of devices in inventory prescribed to residents) and each participant’s adherence to use of the device, which was automatically measured by the smartbelt and reported in the companion app in terms of daily wear hours and compliance (defined as the number of days the device was worn by the resident for at least one hour divided by the total number of days the resident was prescribed to wear the device). Device performance was also to be assessed by monitoring for incidents of serious hip-impacting falls incurred by residents while prescribed to wear the device, whether the airbag deployed during the fall, and whether any major hip injuries were sustained from the fall. Lastly, incidents of airbag deployment which did not occur during serious hip-impacting falls were to be reported.

## III. Results

**FIGURE 3.**
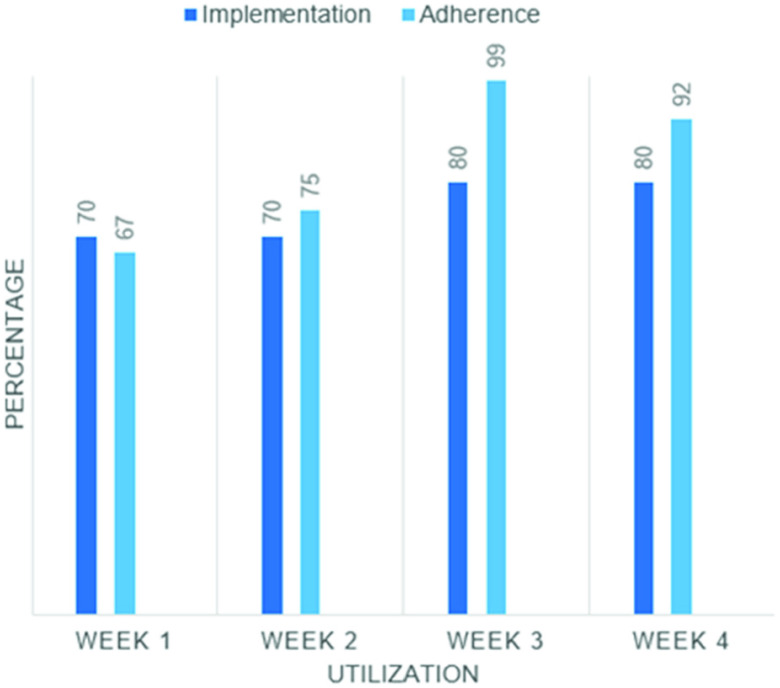
Smartbelt implementation (% of inventory prescribed to residents) and adherence (% of days worn for at least one hour divided by the number of days prescribed to wear the device, averaged across all participants) during the first month of implementation.

Within the first 4 weeks of initiation at the center, 80% of smartbelt inventory was implemented, and participants achieved consistent adherence with compliance averaging above 80%. (See Figure 3.) As it was recognized that there were more candidates in the center that could benefit from the device, the inventory was doubled to 20 smartbelts 4 months after initial implementation. Over a time span of 23 months, a total of 35 residents were consented to participate and wore the smartbelt over various wear intervals as per their physician’s order, amounting to an average of 6.4 wear hours per day (average per resident ranged from 0.9 to 12.9 hrs/day) and a total of 23,886 wear hours from April 2019 – February 2021.

Within the first 10 months of smartbelt implementation at the center, 5 residents wearing the device experienced serious hip-impacting falls which triggered airbag deployments. These falls occurred in the facility with accompanying alerting from the smartbelt to caregivers for attention. Each fall initiated the standard procedures for fall assessment and incident reporting, and in all cases, residents were found to have sustained no major injury with no need for an emergency department visit or hospitalization. Incident reports indicated that each fall required minor follow-up care and each resident immediately returned to their daily activity as previously performed. The number of falls with major injury for the facility decreased by 60% when compared to the same timeframe prior to implementation. While pandemic-related lockdown measures initially impaired smartbelt usage after the first 10 months, in the 23-month time period after implementation, 6 total falls with smartbelt airbag deployments occurred with no associated hip injuries, emergency department visits or hospitalizations.

Descriptions of the fall risk factors for each of the 6 participants who experienced falls with airbag deployment are provided in Table 1, and fall events are summarized in Table 2. Fall mitigation strategies common to all participants (in addition to use of the smartbelt) were based upon standard of care and included medication review, environmental assessment, and physical therapy evaluation and treatment. Summarized below are additional fall mitigation measures and the fall scenario for each incident as reported by staff at the center:

**TABLE 1:** Fall Risk Factors for Residents Wearing Smartbelt who Fell With Airbag Deployment

Fall Risk Factors	Resident 1	Resident 2	Resident 3	Resident 4	Resident 5	Resident 6
Fall in past year	Yes	No	2 or >	2 or >	2 or >	2 or >
Unsteady walking	Yes	Yes	Yes	Yes	Yes	Yes
Poor balance	Yes	Yes	Yes	Yes	Yes	Yes
Leg weakness	Yes	Yes	Yes	Yes	Yes	Yes
Incontinence	Yes	Yes	Yes	Yes	Yes	Yes
Altered sensation in feet	No	No	Yes	No	No	No
Dementia	Yes	Yes	Yes	Yes	Yes	Yes
BIMS score *	3	2	3	3	0	0
Medications for sleep/mood	Yes	Yes	Yes	Yes	Yes	Yes

^*^ The BIMS (Brief Interview for Mental Status) is a cognitive status screening tool used within the MDS 3.0 to capture cognitive screening. Scoring ranges from 00 – 15 with a score of 13–15 indicating cognitively intact, 08–12 indicating moderate cognitive impairment and a score of 00–07 indicating severe cognitive impairment. [14]

**TABLE 2:** Summary of Fall Event Details for Residents Wearing Smartbelt who Fell With Airbag Deployment

Resident	Gender	Age	Fall scenario	Injury reported
1	Female	87	Walking in hallway and grabbed rolling cart	None
2	Female	88	Unassisted rise from wheelchair	None
3	Female	94	Attempted self-transfer from wheelchair to bed	None
4	Female	83	Walking and lost balance (motion data analysis)	None
5	Female	91	Unassisted rise from chair in dining room	None
6	Female	78	Walking from bed to restroom at night	Elbow scrape
Average		86.8		

87-year-old female resident (“Resident 1”) – additional fall mitigation strategies included use of a pressure alarm on bed and wheelchair, and use of a rolling walker. Early in the morning hours, the resident walked out of her room into the hallway with no assistive device wearing the smartbelt. She grabbed onto a medication cart which then rolled, causing the resident to lose her balance and fall towards her right side. The irrecoverable fall motion caused the smartbelt airbags to deploy prior to her hip striking the floor. A nurse sitting at the end of the hall witnessed this fall. The smartbelt triggered alarms that the fall and airbag deployed. The resident was immediately approached and given attention for injury assessment by the staff nurse. The resident had no complaint of pain and was assisted to her feet and walked back to her bed. The resident’s son expressed his gratitude and sense of assurance for having the device on his mother.

88-year-old female resident (“Resident 2”) – additional fall mitigation strategies included use of a pressure alarm on bed and wheelchair, and use of a wheelchair. In the early evening, the resident experienced a fall onto the floor while attempting to rise unassisted to standing from being seated in her wheelchair while wearing the smartbelt. The smartbelt detected an irrecoverable serious hip-impacting fall motion, deployed the airbags and triggered alarms to bring attention. Nurses found the resident on the floor with the wheelchair behind her. The nursing staff completed a physical assessment including checking for range of motion. No injuries were reported, and the resident had no complaints of pain or discomfort. The resident was helped to her feet and returned to her wheelchair.

94-year-old female resident of memory care unit (“Resident 3”) – additional fall mitigation strategies included use of a wheelchair. In the early evening, the resident fell during an attempt to self-transfer from her wheelchair to her bed while wearing the smartbelt. The smartbelt detected an irrecoverable serious hip-impacting fall motion, deployed airbags and sent alerts of the fall. The nursing staff discovered the resident on the floor of her room, provided a physical assessment with no injuries reported and helped the resident to her bed.

83-year-old female resident (“Resident 4”) – no additional fall mitigation strategies. In the early morning hours, an unwitnessed fall occurred while wearing the smartbelt in her room. The smartbelt detected an irrecoverable serious hip-impacting fall motion, deployed airbags and sent alerts of the fall. Nursing staff reported no injury following physical inspection. Post-hoc analysis of motion data captured by the smartbelt indicated a distinct pattern of gait with increasing postural sway prior to the fall, suggesting that the resident attempted to walk from her bed across the room prior to losing her balance and falling onto her side.

91-year-old female resident of memory care unit (“Resident 5”) – additional fall mitigation strategies included use of a pressure alarm on bed and wheelchair. One morning, the resident stood up from the memory unit dining room chair and fell. The smartbelt deployed airbags prior to the individual striking the floor and sent alerts that the fall had occurred. The resident was immediately given attention by nursing staff for physical assessment and found to have no injuries. Family members expressed to the facility team that they were very pleased that she sustained no injuries from the fall.

78-year-old female resident (“Resident 6”) – participant had a past medical history of Parkinson’s Disease, low BMI and frequent falls who was on hospice care in addition to the fall risk factors noted above. Additional fall mitigation strategies included use of a pressure alarm on bed and wheelchair. In the very early morning hours, an unwitnessed fall occurred while wearing the smartbelt in her room. It was believed that the resident was attempting to make her way to the restroom from her bed, lost her balance and fell sideways very quickly onto the floor. The smartbelt deployed the airbags and sent alerts to the care team. Upon assessment, a minor injury of an elbow scratch was discovered. She was helped to her feet and returned to bed. The Memory Support Director in the unit reported her belief that the smartbelt “saved the resident’s hip.”

During the 23-month time period after implementation, there were 2 incidents of airbag deployment not related to a serious hip-impacting fall. The first incident occurred when a participant with dementia became agitated, wriggled out of the smartbelt, and tossed the device across the room. The second incident involved a participant, described by staff as being a “fidgeter,” slipped the device past their hips, leading to airbag deployment as the device fell to the floor. Neither of these incidents resulted in participant injury. Lastly, staff at the center did not report any instances of participants sustaining a fall which resulted in a major hip injury.

## IV. Discussion

To date, the authors are unaware of any previous published case series examining the real-world performance of wearable airbag technology for mitigating fall injuries in older adults at risk of fall injuries. Previously studied fall injury interventions within this population have focused on the use of wearable passive hip pads or environmental modifications, such as compliant flooring or bedside fall mats. However, while hip pads have demonstrated clinical efficacy when worn during the fall [11], [12], widespread adoption has been limited due to poor adherence [13] Similarly, the adoption of compliant flooring has been limited due to the relatively large investment necessary for implementation.

Successful implementation and adherence to use of the smartbelt was attributed to the recognition of a site “champion” in leading the clinical team in candidate identification and device initiation. Other aspects of implementation that supported successful adoption included the embedding of device usage into the established standard of care for fall risk management programming of the center and a center staff-wide education provided by the sponsor for device introduction. Even so, it was noted by staff within the center that not all residents were amenable to wearing the belt, even though they met the screening criteria. Although most of the participants had dementia, the most common cause for refusal cited by staff was dementia-related anxiety or confusion regarding the purpose of the device.

To validate the impact attenuation capability of the smartbelt, fall motion data stored on the smartbelt was retrieved and analyzed post-hoc for two of the fall events deemed to be typical of injurious hip-impacting falls (Resident 1 and Resident 5). Reconstruction and analysis of the descending-phase of the falls revealed estimated peak impact velocities of 3.1 m/s and 2.0 m/s for Resident 1 and Resident 5, respectively. As previously reported by Choi et. al., a kinematic analysis of video-captured falls experienced by older adults in a long-term care facility observed that injurious falls had impact velocities which averaged 2.0 m/s. Reference [15] It can thus be inferred from the estimated peak velocities that these fall events could have been injurious if not for airbag intervention to attenuate ground impact forces.

Limitations of this study include its design as a case series, which does not allow a comparison to be made between the effectiveness of the smartbelt intervention and the current standard of care for fall injury mitigation previously in use within the center. Despite this limitation, as noted previously, the center did report a 60% reduction in the overall number of falls with major injury as compared to a similar period prior to implementation. A further limitation relates to the inability to accurately assess smartbelt adherence during the height of the COVID-19 pandemic. As noted above, the pandemic began in the midst of the study and warranted infection control using lockdown measures (i.e. room confinement and quarantine), which appropriately took priority over fall injury mitigation and greatly disrupted usage of the smartbelt.

## V. Conclusion

Given the scale, severity, and increasing rate of hip fractures suffered by older adults, innovative solutions for fall injury mitigation are desperately needed as an adjunct to the standard of care for fall management. Wearable technologies such as the smartbelt, designed to intervene during serious hip-impacting falls by deploying impact-attenuating airbags, could provide another option for caregivers and healthcare professionals to consider, particularly in long-term care settings with a relatively large population of older adults at risk of injurious falls. Real world evidence of falls with airbag deployment and analysis of fall motion data suggests the feasibility of employing wearable impact-attenuation technologies within these older adult care settings. Future controlled studies are warranted to further establish the efficacy of these new technologies as an adjunct to the standard of care for older adults at risk of injurious falls.
